# LONG-DISTANCE CAREGIVERS’ SATISFACTION AND CHALLENGES WITH FORMAL CARE PROVIDERS

**DOI:** 10.1093/geroni/igz038.2058

**Published:** 2019-11-08

**Authors:** Francesca Falzarano, Jillian Minahan, Verena Cimarolli, Amy Horowitz, Danielle Jimenez

**Affiliations:** 1 Fordham University, Bronx, New York, United States; 2 LeadingAge LTSS Center @UMass Boston, Washington, District of Columbia, United States; 3 Fordham University, New York, New York, United States

## Abstract

The purpose of this study (N=304) was to identify differences in LDCs’ experiences with their care recipient’s (CR) formal care providers (FCPs) among four LDC groups based on CR dementia status and residential setting (community/nursing home [NH]). Results show that LDCs of CRs without dementia living in a NH are less likely to be satisfied with information/communication provided by FCPs compared to LDCs of CRs with dementia in the community. FCP-related challenges were significantly greater among LDCs of CRs in a NH, with or without dementia, compared to LDCs of CRs without dementia living in the community. A significantly greater proportion of LDCs of CRs living in a NH, with or without dementia, reported dealing with inadequate care as a challenge compared to LDCs of CRs with dementia living in the community. This highlights LDCs’ unique experiences related to FCPs based on differences in CR dementia status and residential setting.

